# Kinetic analysis of an anion exchange absorbent for CO_2_ capture from ambient air

**DOI:** 10.1371/journal.pone.0179828

**Published:** 2017-06-22

**Authors:** Xiaoyang Shi, Qibin Li, Tao Wang, Klaus S. Lackner

**Affiliations:** 1 Department of Earth and Environmental Engineering, Columbia University, New York, NY, United States of America; 2 College of Aerospace Engineering, Chongqing University, Chongqing, China; 3 State Key Laboratory of Clean Energy Utilization, Zhejiang University, Hangzhou, Zhejiang, China; 4 School of Sustainable Engineering & Built Environment, Arizona State University, Tempe, AZ, United States of America; Beihang University, CHINA

## Abstract

This study reports a preparation method of a new moisture swing sorbent for CO_2_ capture from air. The new sorbent components include ion exchange resin (IER) and polyvinyl chloride (PVC) as a binder. The IER can absorb CO_2_ when surrounding is dry and release CO_2_ when surrounding is wet. The manuscript presents the studies of membrane structure, kinetic model of absorption process, performance of desorption process and the diffusivity of water molecules in the CO_2_ absorbent. It has been proved that the kinetic performance of CO_2_ absorption/desorption can be improved by using thin binder and hot water treatment. The fast kinetics of P-100-90C absorbent is due to the thin PVC binder, and high diffusion rate of H_2_O molecules in the sample. The impressive is this new CO_2_ absorbent has the fastest CO_2_ absorption rate among all absorbents which have been reported by other up-to-date literatures.

## 1. Introduction

As the announcement of Intergovernmental Panel on Climate Change (IPCC), the CO_2_ emissions will rise between 48 and 55 Gt/yr by 2050, with the energy demands of 40% to 150% increase[1]. Atmospheric CO_2_ will be ranging from 535 to 983 parts per million (ppm) by 2100, roughly double current value, 406 ppm. CO_2_ concentration increment leads to a global mean temperature change from 1990 to 2100 of between 1.4^°^C and 6.1^°^C[2]. The significance and urgency of the development of CO_2_ capture from ambient air has been discussed elsewhere[3–6].

**Fig 1:**
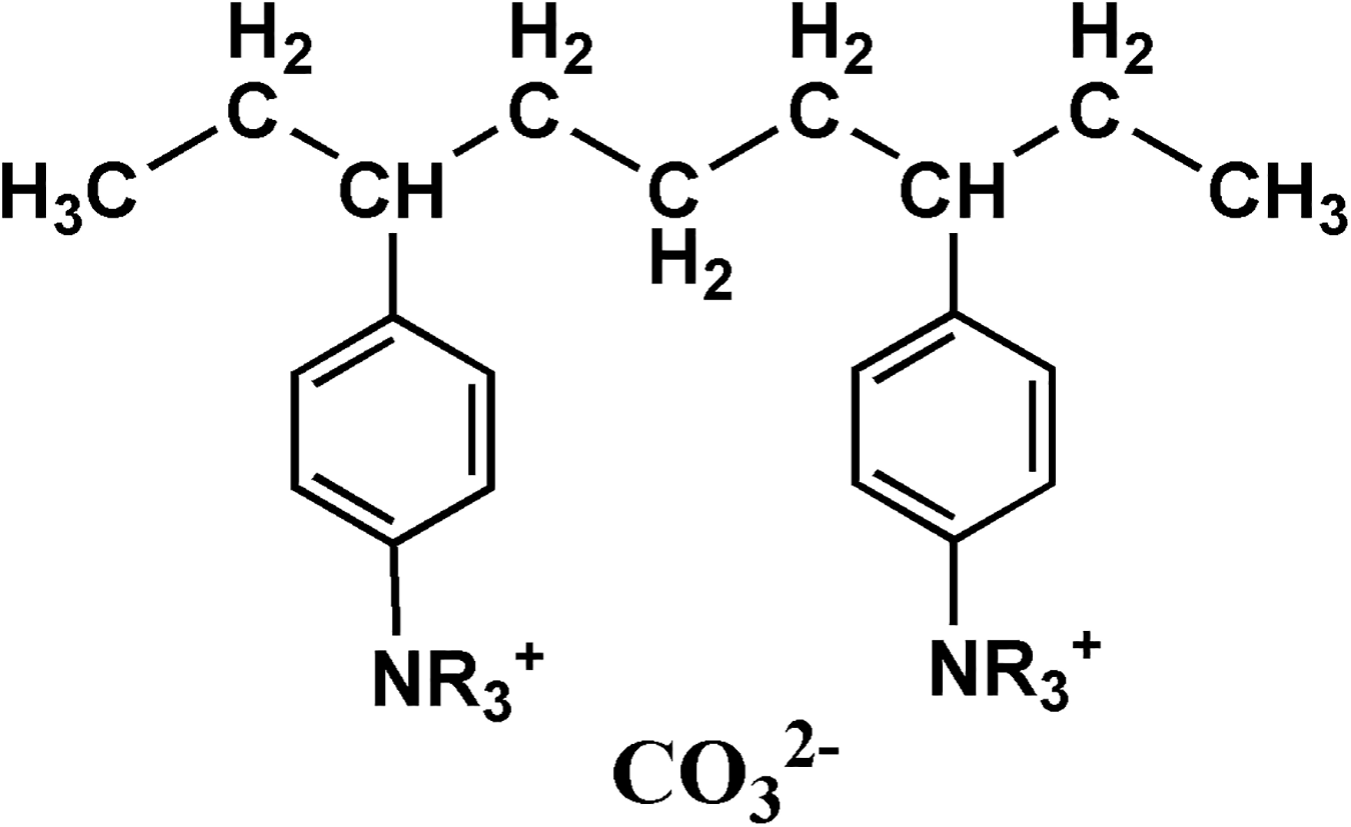
Chemical structure of ion exchange resin containing two side chains. The exchanged anion is CO_3_^2-^.

In order to compensating for CO_2_ emission to ambient air, a moisture-swing sorbent for CO_2_ capture from ambient air was proposed[7], which provides a novel approach to absorb CO_2_ in dilute streams. The moisture-swing CO_2_ absorbent is an anion exchange resin[8–14] (IER). IER acts like a strong base, analogous to NH_4_^+^, where each hydrogen has been replaced by an organic carbon chain attached to a polymer matrix. The chemical structure is shown in Fig 1. The isothermal[15, 16] and kinetic[13, 17] performance of the resin-based sorbent have been revealed systematically. The novel principle of the CO_2_ absorption/desorption process over IER was well illustrated and clarified[8, 18]. The reason is that reduction of the number of water molecules presenting in the pore space promote the hydrolysis of CO_3_^2-^ to HCO_3_^-^ and OH^-8^. This phenomenon enables a nano-structured CO_2_ absorbent possible to capture CO_2_ spontaneously in the ambient air when the surrounding is dry, while releasing it when exposed to moisture[8, 9]. In the dry condition, a CO_3_^2-^ ion splits a H_2_O molecule to form a HCO_3_^-^ and a OH^-^ ion which both bind tightly to their respective NR_4_^+^ cations. OH^-^ ion absorbs CO_2_ even at a low partial pressure of CO_2_. This results in a CO_2_-loaded state which is entirely HCO_3_^-^. In the wet condition to regenerate the full-loaded absorbent, each two HCO_3_^-^ ions react to produce a CO_3_^2-^ ion, a H_2_O and a CO_2_. The released CO_2_ in the wet condition can be collected collectively. The underlying mechanism has been revealed by Shi[9] and the phenomenon can be applied to a series of counter-intuitive chemical reactions which is related to the hydrolysis of basic ions[10]. Meanwhile, the discovery opens a new approach for the technology of gas separation.

For these conditional CO_2_ capture methods, like thermal-swing CO_2_ absorbents[19], increasing absorption capacity is a significant task due to the high cost (the regeneration process consumes heat) on the absorbent regeneration. However, for the novel water-driven CO_2_ absorbent, kinetics improvement is a more interesting factor due to the low cost (the regeneration process consumes water) of the regeneration part[17]. The energy consumption and cost can be reduced significantly according to improving the absorption rate of the water-driven CO_2_ sorbent. The objective of this study is to propose a new moisture-swing CO_2_ sorbent (P-100) by using ion exchange resin (IER) as a functional group and polyvinyl chloride (PVC) as a binder. The kinetic characterization of the new CO_2_ sorbent has been enhanced significantly comparing to sorbent I-200[15], which is manufactured by Snowpure LLC, California. The preparation process of this new sorbent is introduced first, and the analysis of kinetic performance is presented next based on the studies of material structure, CO_2_ absorption/desorption process and water diffusion experiments.

## 2. Materials and preparation process

### 2.1 Materials

A heterogeneous ion-exchange material in a flat sheet was prepared in this study. The material includes: 1) ion exchange resin (IER)[7]. The IER is composed of a polystyrene backbone with attached quaternary amine ligands. 2) Polyvinyl chloride (PVC). PVC is a widely produced synthetic plastic polymer which was used as a binder. 3) Tetrahydrofuran (THF). THF is an organic compound with the formula (CH_2_)_4_O which was employed as a solvent to mix IER and PVC.

### 2.2 Preparation of anion-exchange sorbent

The heterogeneous CO_2_ absorbent was prepared by using dip-coating technique[20]. First, the IER particles were grinded in a ball mill and then filtered using a mesh with 44~74 micrometer openings. Then, PVC was dispersed into THF in a glass reactor and stirred mechanically for more than 5 hours. The weight ratio of PVC to THF is 1:20. Next, powdered resin particles (44~74um) were added into the mixture of PVC and THF. The mechanical stirrer stirred vigorously at room temperature for 30 minutes to mix IER and PVC uniformly. The IER to PVC weight ratio is 1:1 and the total solid to THF ratio is 1:10 (w/v)[21]. After completed mixing, the dip coating method was conducted by using dry clean glass plate. The thickness of the produced membrane was 100 micron. The membrane was dried in the ambient air at temperature 25°C for 30 min, and then was immersed in distilled water. Last, three absorbent samples were treated by different temperatures of water for 48 hours (25°C water (P-100-25C), 50°C water (P-100-50C), 90°C water (P-100-90C)). I-200 sample was only treated by 90°C water as a reference. Three P-100 samples and an I-200 sample, containing the same load of IER, were immersed in 1.0 M sodium carbonate solution for 2–4 hours[15]. Samples were washed 4–5 times, and then washed by plenty of deionized water (DI water) to flush away the sodium carbonate solution residues on the samples. Afterwards, the samples were ready-to-use.

### 2.3 The absorption capacity of CO_2_ sorbent

The Mohr method was used to determine the effective ion charge density *ρ*_*c*_ of the IER. The *ρ*_*c*_ is 1.58 mol/kg. CO_2_ capacity *Q*_*est*_ is 17.69 L/kg, which was estimated by *ρ*_*c*_ at standard condition. The CO_2_ capacity *Q*_∞_ was also measured by experiments under the condition of 1000 ppm CO_2_ partial pressure. The value of *Q*_∞_ is 16.4L/kg. The effective charge density and CO_2_ capacity of the absorbent can be both enhanced according to increasing the weight ratio of IER to PVC during the sample preparation process.

## 3 Experimental methods and models

### 3.1 Absorption experiment

**Fig 2:**
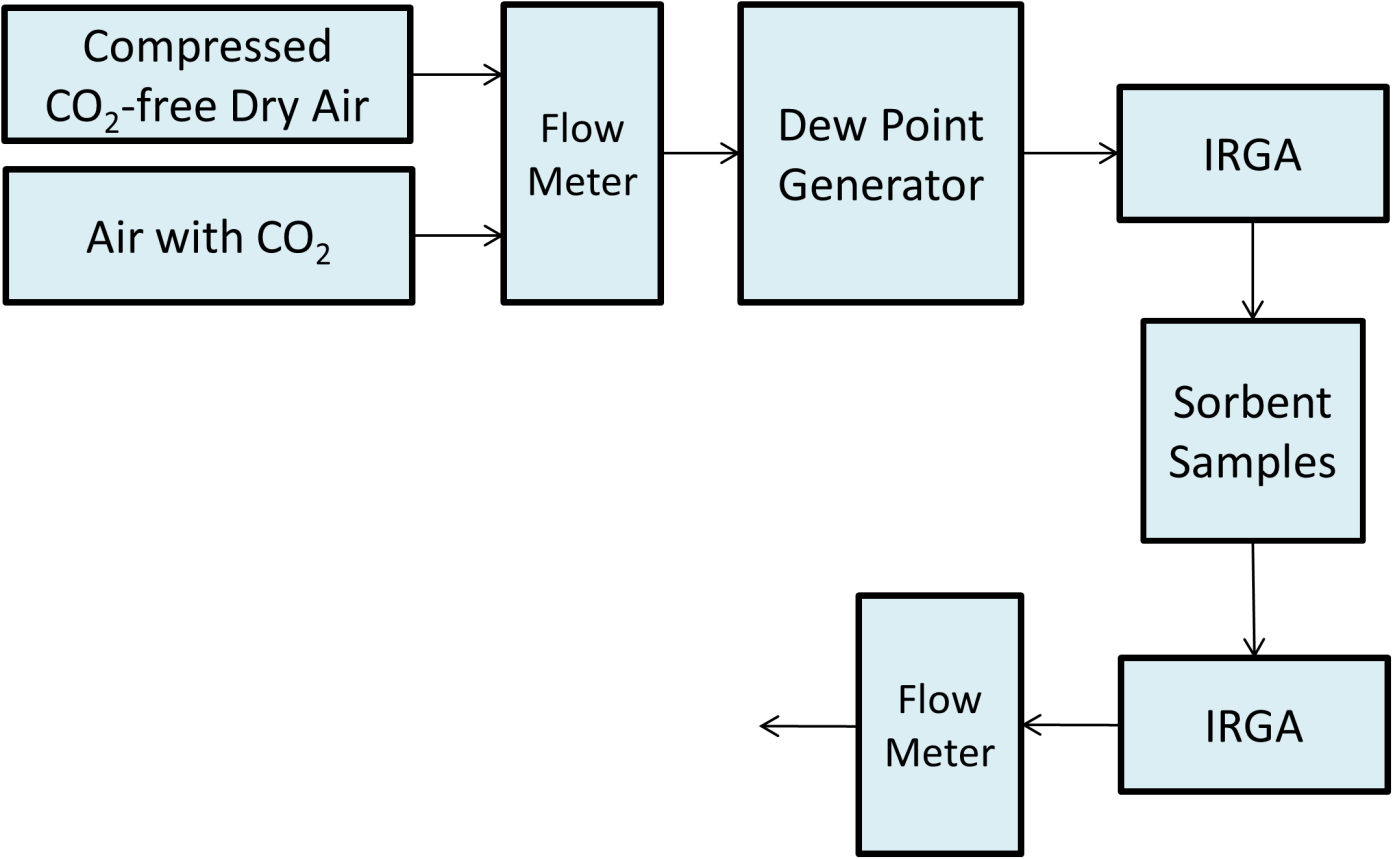
Schematic of experimental device. The CO_2_ concentration can be tracked at inlet and outlet of the chamber of sorbent sample. The amount of absorbed CO_2_ can be calculated by measuring the CO_2_ concentration change with time. Dew point generator can control the water vapor concentration in the system.

The experimental device with humidity control was set up to measure the half-time (the time when the absorbent reaches half of its capacity) of the moisture-swing CO_2_ absorbents. A layout of the device is shown in Fig 2. The CO_2_ concentration change was measured by two infrared gas analyzers (IRGA). Measurements were recorded once per second. The wet and fresh samples (each sample contains 0.30g IER) were put into a sealed chamber one by one and flushed with 2L/min CO_2_-free dry air. The water concentration in the air at outlet was monitored to determine whether the samples were sufficiently dried. (The water concentration in the CO_2_-free dry air at inlet and water concentration in the CO_2_-free dry air at outlet were same, when samples had been sufficiently dried. The water concentration was about 2% relative humidity,). The 1 L/min air containing 400 ppm CO_2_, went through a dew point generator (MODEL LI-610). The air contained 30% relative humidity and flowed over all sorbent samples. Entire absorption process would last until CO_2_ concentrations were same at inlet and outlet with 1% error.

### 3.2 Desorption experiment

**Fig 3:**
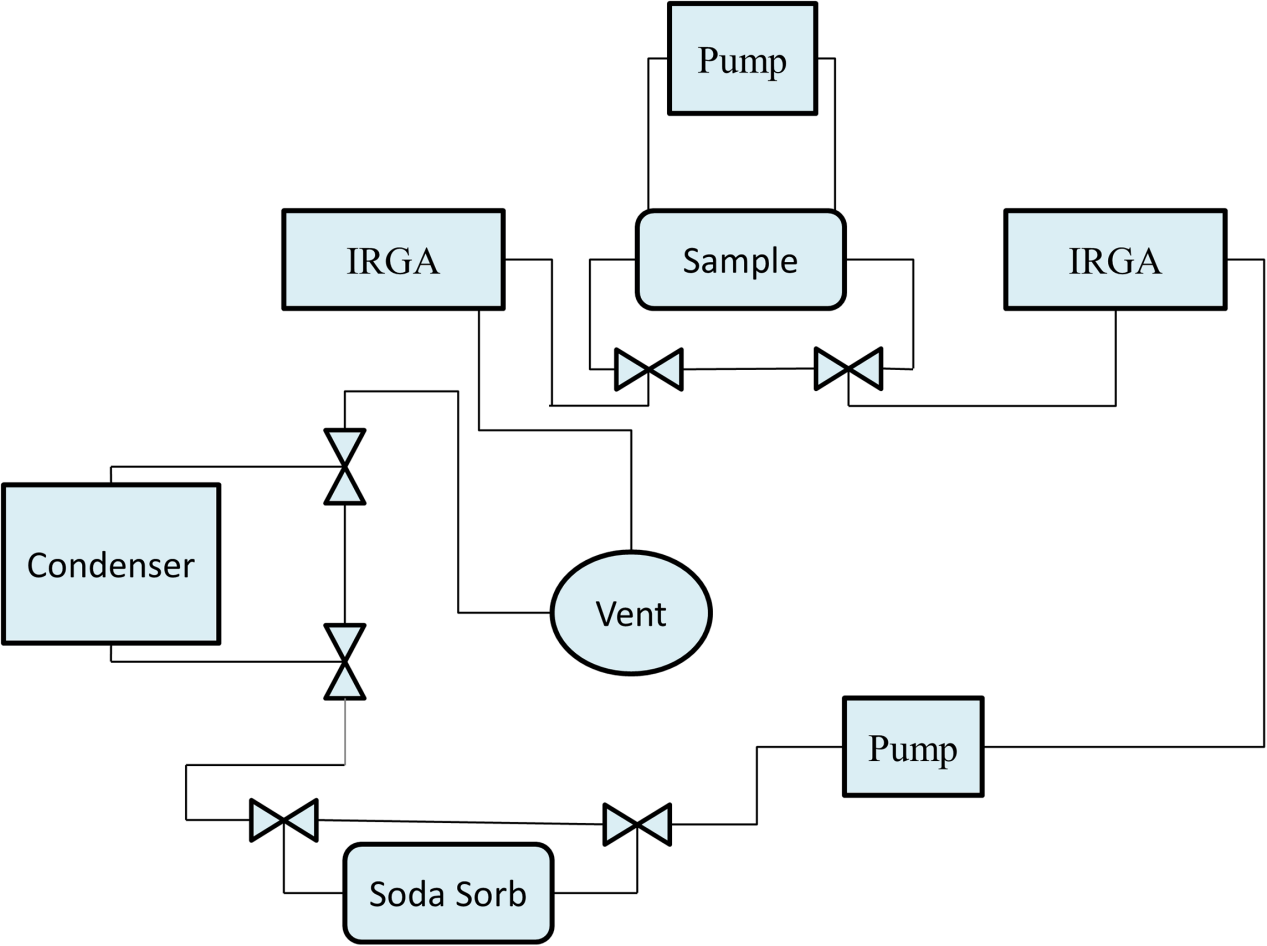
The schematic of the experimental device. The total amount of CO_2_ in the sample and in the gas volume is constant. The process of CO_2_ absorption/desorption can be identified in the experimental device.

The absorbed CO_2_ by absorbents will be released when the absorbents are exposed to a high humidity or liquid water. Meanwhile, the absorbents continue to absorb water vapor from air when they are put into a higher humidity until the system reaches to an equilibrium state. The absorbed water molecules increase the weight of the absorbents. Here, the desorption experiment is to analyze the kinetic characteristics of the absorbents by studying the equilibrium time of the process of H_2_O absorption and CO_2_ desorption. The diffusion coefficients of water molecules in the CO_2_ absorbents were derived by calculating the weight change of the samples. The diagram of the experimental device is shown as Fig 3

The total amount of CO_2_ in the closed-loop experimental device is constant. We can track the amount of absorbed/desorbed CO_2_ by measuring the CO_2_ concentration in the air. The device can control the water concentration in the air by heating or cooling the condenser. The weight change of the absorbents can also be measured by weight scale in the device.

Four samples (25°C-water-treated P-100, 50°C-water-treated P-100, 90°C-water-treated P-100, and 90°C-water-treated I-200) containing the same resin load, were firstly exposed to pretreated dry air (dew point -18°C) for two hours to be fully dried and loaded. Next, put the full-loaded samples into experimental device (as Fig 3) subsequently and increased the humidity level to 15°C. The increase of sample weight and CO_2_ concentration in the experimental device were measured separately. The increase of the sample weight was mainly due to the water absorption on the samples. A humidity controller was employed to ensure a constant humidity in the chamber by PID control. CO_2_ concentration change was recorded every second by infrared gas analyzers (IRGA).

### 3.3 Absorption kinetics of sorbent

The absorption characteristics of P-100-90C sorbent was depicted by Lagergren pseudo first-order model[22], which has been most frequently employed to present absorption dynamic process under various conditions.

$$\frac{dq}{dt}=k(q_{e}-q)$$ (1)

*q* is absorption quantity at time *t*, *k* is constant number, *q*_*e*_ is equilibrium isotherm absorption capacity. Integrating Eq 1 with boundary conditions (a) *t* = 0, *q* = 0; (b) *t = t*, *q = q*_*e*_

$$q=q_{e}(1-e^{-kt})$$ (2)

## 4 Results and discussion

### 4.1 Absorbent structure analysis

**Fig 4:**
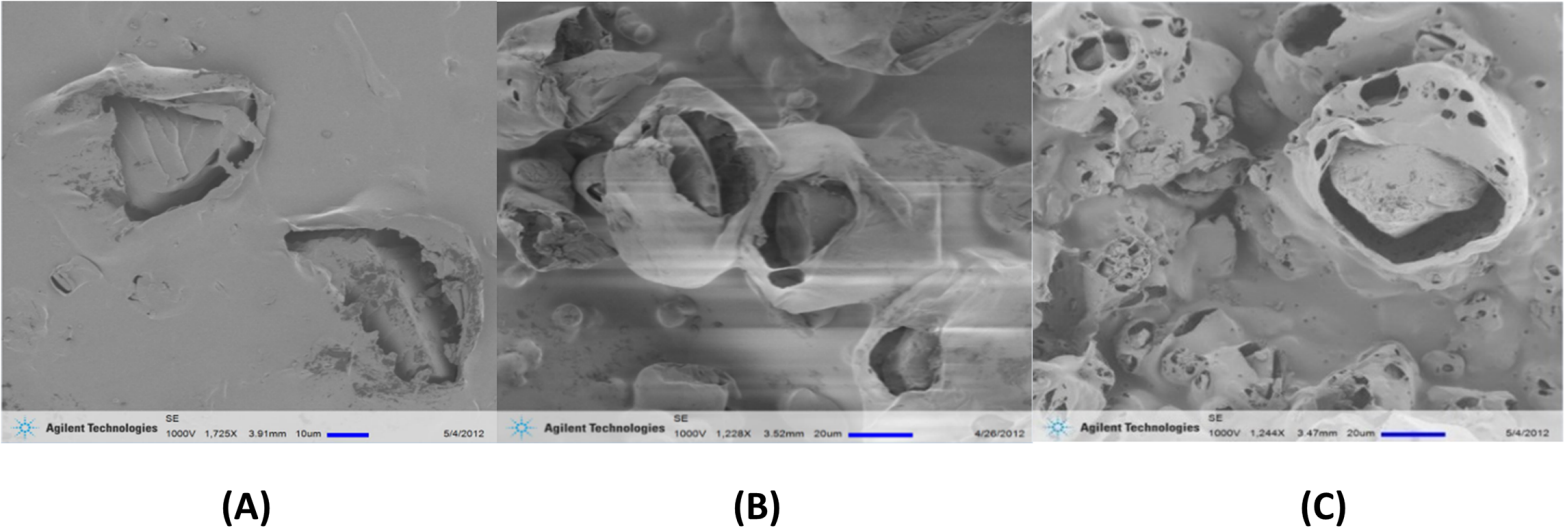
SEMs of P-100 sorbents treated with different water temperatures. (A) 25^°^C-water-treated sample P-100-25C, (B) 50^°^C-water-treated sample P-100-50C, (C) 90^°^C-water-treated sample P-100-90C.

The structures of the P-100 sorbents, which were treated by different-temperature hot water, were studied by scanning electron microscope SEM (Agilent Technologies, SE 1000V) as shown in Fig 4. Obviously, hot water treatment results in the formation of narrow cavities between anion exchange particles and PVC matrix, as well as larger amount of pores in PVC materials.

Micro-structure schematic of P-100 sorbent can express the percolation structure of P-100 sorbent. After the treatment of 90^°^C hot water, some small islands of interconnected particles appear and these connections form extended pathways. More and more IER particles are connected by channels if the connections keep growing. The chance of the appearance of percolation threshold can increase the rate and range of gas diffusion inside the sorbent. According to the observation of SEM, much more resin particles in the new sorbent P-100 are exposed into air than the ones held by I-200[15] because of the thinner thickness of membrane and the more continuous channels inside P-100. Moreover, percolation threshold may further promote the conduction level between surrounding air and resin particles. Therefore, treating P-100 by hot water may promote IER particles to be exposed to ambient air, thereby further improving the performance of the CO_2_ absorbent.

### 4.2 Absorption half-time

The kinetic characteristics are significant factors for moisture-swing CO_2_ capture sorbent. Absorption kinetics of the sorbent are determined by mass diffusivity in the materials, heat transfer into and out of the pores, and intrinsic chemical reaction rates[17, 23, 24]. As a preliminary assessment for the CO_2_ absorption kinetics, absorption half time is an assessment factor^23^ to evaluate the absorption rate of CO_2_ absorbent. The absorption half time is expressed by Eq 3:

$$T_{half-time}=\frac{T_{{CO}_{2}}}{2}=T_{H_{2}O}+T_{reaction}$$ (3)

$T_{{CO}_{2}}$ is the time for CO_2_ absorption by sorbent from fresh-empty status to full-loaded status, $T_{H_{2}O}$ is the absorption time of water on sorbent; *T*_*reaction*_ is the time of intrinsic chemical reaction. The half time of sample P-100-25C, P-100-50C, P-100-90C, and sample I-100 was measured by experimental device, shown as Fig 2.

**Fig 5:**
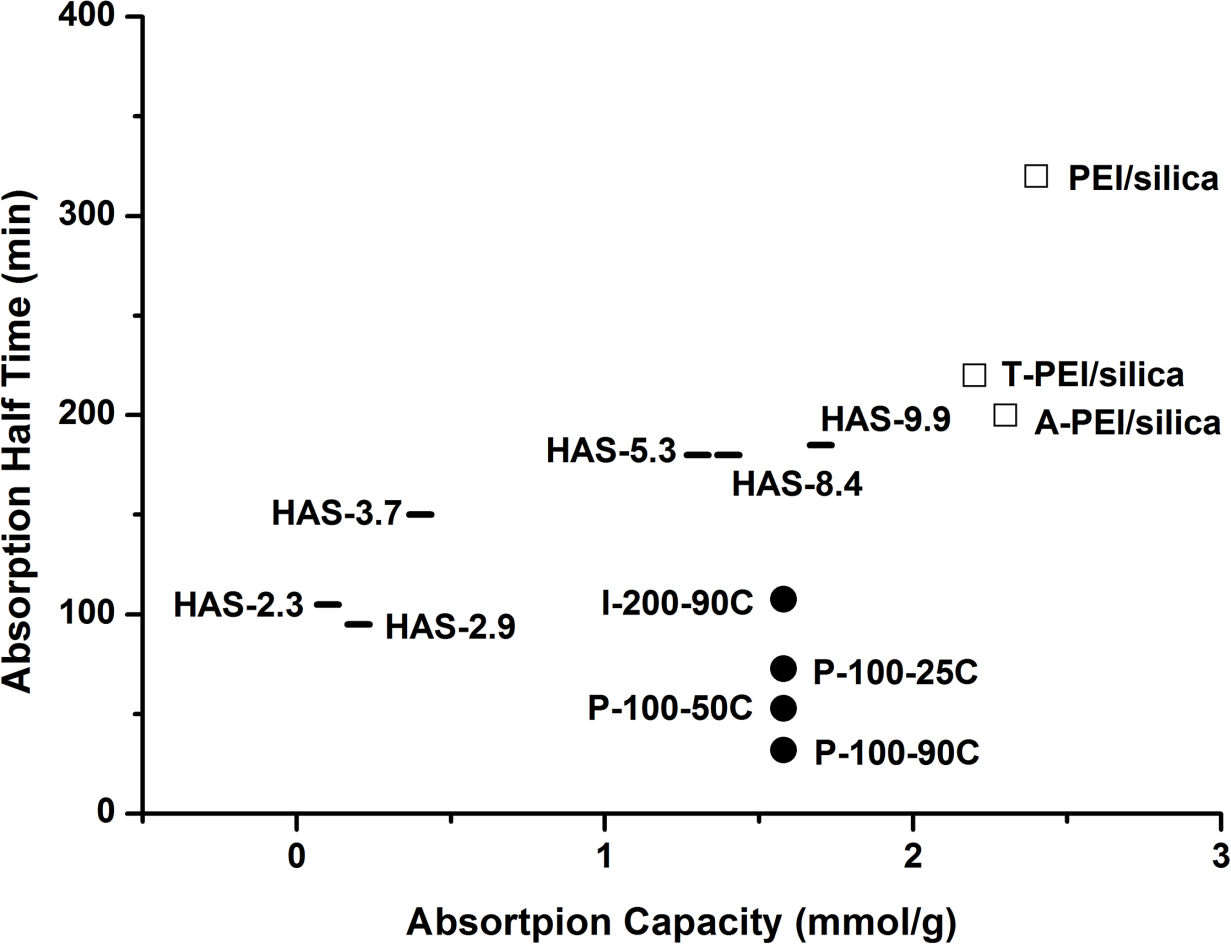
Comparison of CO_2_ absorption half times and capacities of different sorbents. Hyperbranched aminosilica (HAS) with different amine loading (-), PEI/silica materials (□), and moisture-swing Ion Exchange Resin (IER) (●). The number of half time and absorption capacity of each absorbent has been shown in S1 Table.

Fig 5 displays the half times of the four different samples under simulated air capture condition (30% relative humidity, 400ppm). Other half time of current air capture CO_2_ sorbents[23, 25] in literatures have also been presented.

The sample P-100-90C owns the best kinetic characteristics comparing with the other three moisture-swing CO_2_ sorbents. The 31.8 min half time is also the shortest half time in all air capture sorbents which have been reported by other up-to-date literatures. Obviously, the kinetics of P-100-90C is better than the other two P-100 sorbents, because the hot water treatment enlarges the surface area of IER to be exposed to the ambient air. This leads to a much faster absorption/desorption rate of water molecules in IER than the other two P-100 samples, meaning the smaller $T_{H_{2}O}$ value. The reaction rates of moisture-swing CO_2_ absorbents were estimated similar.

I-200 moisture-swing CO_2_ absorbent was also treated by 90^°^C hot water but still had a longer half time than those of three P-100 samples. The reason is the thickness of I-200 sorbent is 640 microns which is much thicker than the 100 microns thickness of P-100 sorbent. Plenty of time is consumed by water vapor to permeate into I-200 sorbent to contact the inside IER particles wrapped by polypropylene matrix binder.

**Fig 6:**
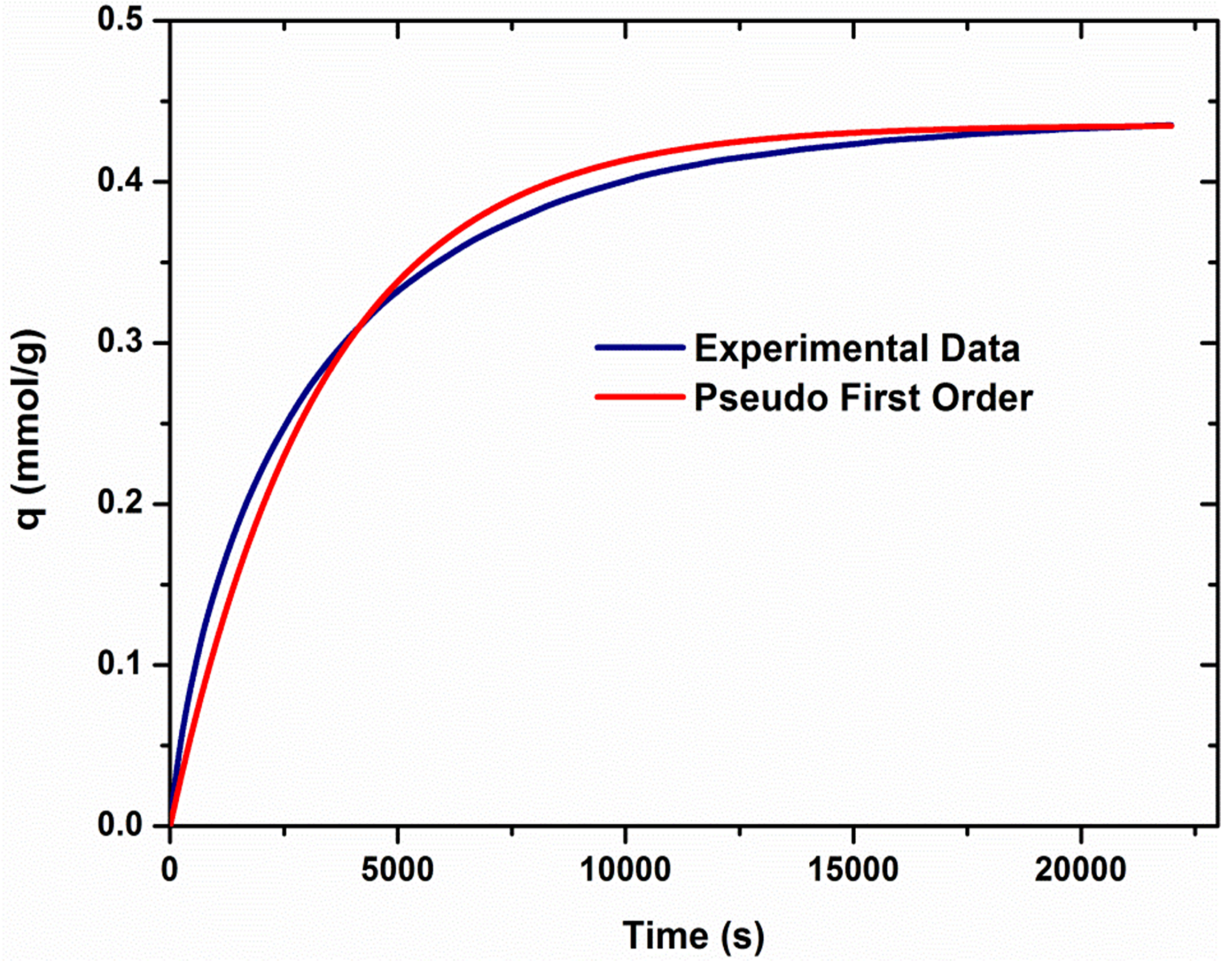
Comparison of kinetic model and experimental data for absorption performance of P-100-90C absorbent.

Fig 6 shows that pseudo first order model as Eq 1 and 2 fits the absorption kinetic data of P-100-90C absorbent with the coefficient of determination 0.98. The amount of absorbed CO_2_ by P-100-90C sorbent was recorded per second, and the *k* values were determined by Eq 2.

### 4.3 Desorption kinetic performance

Moisture-swing CO_2_ absorbent can release CO_2_ back to the air in a wet surrounding^8^. The desorption kinetic characteristics of the moisture-swing absorbents are mainly influenced by the diffusion rates of H_2_O and CO_2_ in sorbent. This study focuses on analyzing the impact of these two factors on the desorption rate.

To determine the diffusion coefficients of water molecules in the four absorbents, the moisture uptake percentage was determined from the equation:

$$Moisture\;Uptake\%=\frac{M_{t}}{M_{d}}\times 100\%$$ (4)

Where *M*_*t*_ is the total amount of water content absorbed by sorbent samples at time *t*, *M*_*d*_ is the original weight of the dry samples. The diffusivity *D* was determined from the slope (*K*) of the initial linear region of the plot of the percentage moisture uptake $\frac{M_{t}}{M_{d}}$ versus $\sqrt{t}$ curve[26, 27].

$$D=\frac{\pi }{16}{\left(\frac{h}{M_{\infty }/M_{d}}\right)}^{2}K^{2}$$ (5)

Where *h* is the thickness of the sample, *t* is exposure time and *M*_∞_ is the maximum moisture gain.

**Fig 7:**
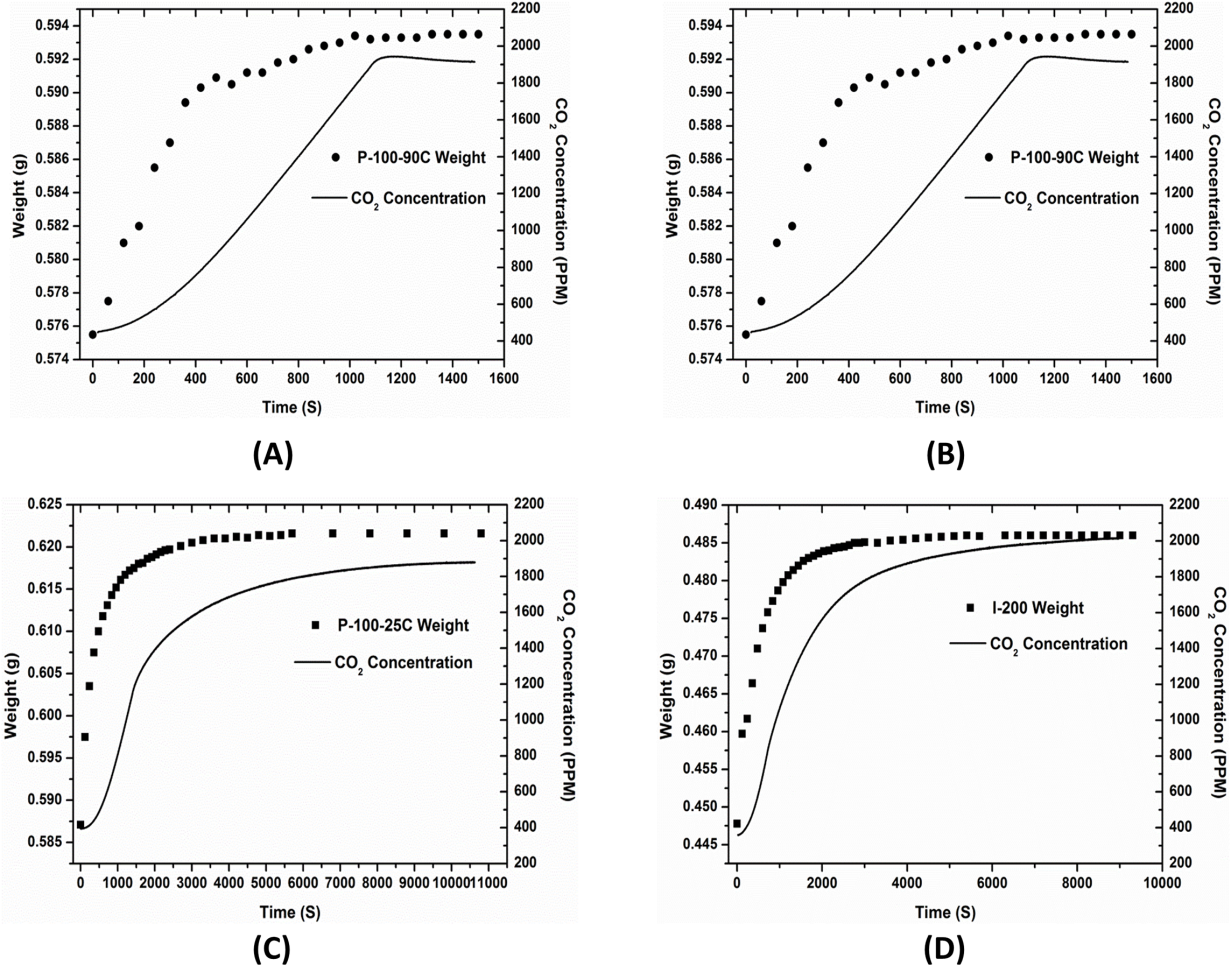
CO_2_ desorption process of four absorbents (A) P-100-90C absorbent, (B) P-100-50C absorbent, (C) P-100-25C absorbent, (D) I-200 absorbent. Left Y-axis is absorbent weight, and right Y-axis is CO_2_ concentration.

Fig 7 illustrates the desorption process of the studied four absorbents. The four samples are comparable because they have the same weight of IER particles about 0.30g. The same weight of IER particles can maintain the equilibrium concentration of CO_2_ is between 1900ppm and 2000ppm under the same condition of dew point 15°C in the experimental device, shown as Fig 3. The samples increasingly absorb water molecules over time, which leads to their weights increase to equilibrium values under a certain water vapor partial pressure. Absorbed water molecules are conducive to desorb CO_2_ from the full-loaded absorbents. The released CO_2_ from absorbent increases the CO_2_ concentration in the experimental device. The coefficient of water diffusion, as well as the equilibrium time of T_w_ (the equilibrium time of absorbed H_2_O by absorbent) and T_c_ (the equilibrium time of desorbed CO_2_ by absorbent) of the four absorbents have been listed in Table 1.

**Table 1:** Diffusion coefficients of water, equilibrium times of water and CO_2_ in four samples.

	D(m^2^/s × E-12)	T_w_ (s)	T_c_(s)	ΔT (s)
(A)P-100-90C	6.503	1020	1086	66
(B)P-100-50C	2.536	1740	2462	722
(C)P-100-25C	1.842	7200	10628	3428
(D)I-200	58.464	6300	8970	2670

D is the diffusion rate of H_2_O; T_w_ is the equilibrium time of absorbed H_2_O by absorbent; T_c_ the equilibrium time of desorbed CO_2_ by absorbent. ΔT = T_c_−T_w_

Comparing Table 1 (A), (B) and (C), the diffusion rate of water is higher when sorbent is treated by higher temperature hot water. The higher diffusion rate is due to larger number of pores in PVC matrix, as well as larger narrow cavities between IER particles and PVC binder. These characteristics greatly promote the diffusion rates of H_2_O and CO_2_ in sorbents. Higher diffusion rate of water can help ions in the absorbents to move more quickly and help desorbed CO_2_ to release back to air more rapidly. For the P-100-90C sorbent, the equilibrium time difference Δ*T* between T_w_ and T_c_ is smallest ΔT = 66s. It means the desorbed CO_2_ diffuses out of the sorbent costing 66s after water reaches to equilibrium in the sorbent. However, ΔT of P-100-25C is as long as 3428s. It means the released CO_2_ is still trapped in sorbent for a certain amount of time.

Comparing (A) and (D), both sorbents had been treated by 90^°^C hot water. Though the diffusion coefficient of water in I-200 is much larger than the one of P-100-90C, the water equilibrium time of P-100-90C is much smaller than those of I-200 due to the different binder materials of two sorbents. The reason is hydrophilic polypropylene binder of I-200 may also contribute to the water diffusion than the hydrophobic PVC binder of P-100-90C. However, the polypropylene of 640 micron thickness costs more time for water diffusion than the 100 micron PVC. The polypropylene binder of I-200 (provided directly by Snowpure LLC, California) is very difficult to be thinned due to the essential characteristics of this polymer.

I-200 also needs longer time for CO_2_ to release back to the ambient air. For the I-200 absorbent, the IER particles of 44~74um diameters are inlayed in polypropylene of 640 micron thickness. The narrow, tortuous and long paths among resin particles and polypropylene are against to CO_2_ diffusion. However, the same IER particles of 44~74um diameters can almost penetrate the 100 micron PVC in P-100-90c sorbent. IER in P-100-90c sorbent can contact atmosphere directly is benefit to desorption performance. Hydrophilic polypropylene binder in I-200 can also attract some absorbed water molecules. This part of water molecules can not contact functional IER particles to release CO_2_.

## 5. Conclusion

This study introduces a new moisture-swing CO_2_ absorbent by employing polyvinyl chloride (PVC) as binder for ion exchange resin (IER). The manuscript analyzes the preparation process, absorbent structure, kinetic model, absorption and desorption characteristics of this CO_2_ absorbent. The CO_2_ absorption rate of this new produced absorbent P-100 is nearly three times as fast as the one of I-200, and also three to ten times as fast as amine-tethered solid CO_2_ absorbents. This fast absorption/desorption rate is with the benefit of a thin bind holder and fast diffusion rate of H_2_O. This new CO_2_ absorbent provides a way of designing a moisture-swing CO_2_ absorbent with a high absorption/desorption rate in the future.

## Supporting information

S1 Table Half time and absorption capacity of CO_2_ absorbent. (DOCX)
