# Supplementary material for: Kinetic analysis of an anion exchange absorbent for CO2 capture from ambient air
Source: PLoS One. 2017 Jun 22;12(6):e0179828. doi: 10.1371/journal.pone.0179828 (PMC5480984; doi:10.1371/journal.pone.0179828)
Supplement: S1 Table — (DOCX) [file pone.0179828.s001.docx]

**Supporting Information**

| Samples | Absorption Capacity (mmol/g) | Half Time (min) |
| --- | --- | --- |
| HAS-2.3 | 1.58 | 31.8 |
| HAS-2.9 | 1.58 | 52.8 |
| HAS-3.7 | 1.58 | 72.7 |
| HAS-5.3 | 1.58 | 107.5 |
| HAS-8.4 | 0.1 | 105 |
| HAS-9.9 | 0.2 | 95 |
| PEI/silica | 0.4 | 150 |
| A-PEI/silica | 1.3 | 180 |
| T-PEI/silica | 1.4 | 180 |
| I-200-90C | 1.7 | 185 |
| P-100-25C | 2.4 | 320 |
| P-100-50C | 2.3 | 200 |
| P-100-90C | 2.2 | 220 |

Table S1 Half time and absorption capacity of CO_2_ absorbent
